# Experiences of faculty and students regarding a locally developed framework for implementing interprofessional education during international electives in Sub-Saharan Africa

**DOI:** 10.1186/s12909-023-04664-9

**Published:** 2023-09-26

**Authors:** Faith Nawagi, Ian Guyton Munabi, Andre Vyt, Sarah Kiguli, Aloysius Gonzaga Mubuuke

**Affiliations:** 1 School of Medicine, Makerere University College of Health Sciences, Kampala, Uganda; 2 School of Biomedical Sciences, Makerere University College of Health Sciences, Kampala, Uganda; 3 Faculty of Medicine and Health Sciences, University of Ghent, Gent, Belgium

**Keywords:** Interprofessional Education and collaborative practice, Framework, Africa, Students, Faculty

## Abstract

**Background:**

Given that there are hardly any comprehensive frameworks to guide institutions on approaches to use as they implement interprofessional education and collaborative practice during international electives, we developed and piloted a framework to address this gap. The purpose of this study, therefore, was to explore the experiences of faculty and students regarding the use of the developed interprofessional education and collaborative practice framework during international electives.

**Methods:**

This was an exploratory qualitative study. The study participants included faculty and students from four health training universities in Africa who participated in the pilot of international electives guided by the framework developed. Deductive thematic analysis was used to analyze the data. The codes were categorized as per the major themes.

**Results:**

The major themes regarding the framework included (1) The Strengths, (2) Weaknesses, (3) Opportunities, and (4) Threats. All participants perceived the framework as useful and appropriate to enable the acquisition of interprofessional education and collaborative practice skills objectives set. The framework’s duration of the elective was seen as a weakness with the need for an increment in the duration. The opportunities the framework presented included: acting as a starting point to structure and implement interprofessional education across various training institutions in Africa, advancing research, and networking opportunities to share the best practices. The main threat included siloed training where the current training curriculum of the students does not have opportunities that allow the students to study with, from, and about each other.

**Conclusions:**

The framework developed to guide the implementation of interprofessional education and collaborative practice during international electives is feasible and enabled students to achieve the interprofessional education and collaborative practice objectives set while appreciating the transcultural similarities and differences in another country.

**Supplementary Information:**

The online version contains supplementary material available at 10.1186/s12909-023-04664-9.

## Introduction

Interprofessional Education (IPE) is one of the key drivers to team-based care which has been evidenced to improve patient outcomes [1]. Furthermore, Inter-professional Education and Collaborative Practice (IPECP) in healthcare can enhance healthcare outcomes through shorter hospital stays, reduced costs, and enhanced productivity among the healthcare providers [2].

Globally, various health professional training institutions are making efforts to incorporate IPE in their curricula, teaching, and learning environments. Internationalization in Health Professions Education (HPE) has also gained momentum demanding innovative ways of learning and exposure [3]. Various institutions globally and in Africa have developed International Electives (IEs) through unilateral, bilateral, and multilateral partnerships between home and host institutions often in different countries [4]. However, students who undertake IEs do so in professional silos rather than in inter-professional teams [5], characterized by the pairing of students with similar disciplines during IEs rotations. International Electives (IEs) are defined as the time of learning where students have a choice on where to learn, and what specialty program they should be learning from [6]. IEs as a form of teaching and learning are part of various health professions curricula [7] [8] with faculty being the key drivers for their effective structuring [9] [7] [8]. These have been documented to enhance the learners’ global perspectives, knowledge and skills, interpersonal and professional development, and positive attitudinal change towards health care [10]. Furthermore, IEs provide a learning platform where IPECP skills can be cultivated especially if offered with a structured approach [11]. They provide students with an opportunity to cultivate cultural humility through exposure to different professional norms in another country which could be transferred to their future practice. A few studies that have been conducted in Africa have described the development of IEs in general and the use of the regional mobility model [12] but without a focus on the IPECP approach. The virtual learning platform has also gained momentum in Africa especially due to the COVID-19 pandemic that encouraged many institutions to embrace online learning [13]. However, little is known about utilizing virtual online learning for IEs and with an IPECP approach.

Efforts have been made by the World Health Organization [14], Interprofessional Education Collaborative [15], and most recently the European Interprofessional Practice and Education Network [16] among others to develop frameworks that elaborate more on the core competencies of IPECP. Despite the relevance of the latter frameworks, it is key to note that IPECP can occur in various learning environments including International Electives (IEs). However, there are hardly any comprehensive frameworks to guide institutions on implementation and approaches to use as they implement IPECP during IEs. Furthermore, given that IPECP-IEs require attachment to another country, a guide for intercultural orientation hardly exists.

To address the above gap, we developed an IPECP-IEs framework as shown in Appendix 1 using a modified Delphi technique to guide institutions on how to implement IPECP-IEs. The framework was piloted in partnership with four African health professional training institutions using a virtual approach and country-specific case studies as the basis for training. The purpose of this study, therefore, was to explore the experiences of faculty and students regarding the use of the developed IPECP-IEs framework during its pilot.

## Methodology

### Study design

This was an exploratory qualitative study conducted among faculty and students at four health professional training institutions in Africa who participated in the pilot of the IPECP-IEs framework developed. Key Informant Interviews (KIIs) were used to collect data from the faculty and student participants. The details of the pilot of the framework are presented in Appendix 1 to illustrate all the steps and approaches used to pilot the framework.

### Study context and setting

The study was contextualized within the context of the African Forum for Research and Education in Health (AFREhealth) in Africa. AFREhealth is an interdisciplinary health professional group that works with ministries of health and training institutions to improve the quality of health care in Africa through research, education, and capacity building [17]. AFREhealth runs an international student elective program in fifteen training institutions. The purpose of these electives is to enhance global exposure for students in various healthcare domains and systems. The electives are managed through partnerships with home and host institutions with direct supervision of the students learning by faculty at the host institution [5]. Using the AFREhealth Elective Program web-based online application system, all students have access to the various elective opportunities and apply for an elective of choice in specific disciplines. Each institution hosts five students lasting six weeks. Out of the fifteen institutions participating in the wider AFREhealth elective program, four institutions were included in this study. These include Makerere University (Uganda), Kenyatta University (Kenya), University of Ibadan (Nigeria), and University of Zimbabwe (Zimbabwe).

These institutions were selected based on the criteria/ justification that they have electives included as part of their curriculum and are in the east, west, and southern parts of Africa and thus a representation of Africa. Furthermore, they use English as their language of instruction and host many students for international electives annually. The four training institutions have electives available at the undergraduate level for students in their clinical years of training. Electives at these four institutions are open at varying times based on the academic calendar. Specific to the AFREhealth elective program, over five students participate in the program per institution per year [12]. Beyond the AFREhealth elective program, about twenty students in each of these institutions participate in International electives [12]. However, all four institutions host more than thirty students per year from other training institutions for International elective programs being offered [12].

### Framework used to implement the IPECP-IEs

The framework was piloted from August – November 2022. This framework was developed using a modified Delphi technique. The framework ( Appendix1) contains sections that provide guidance on; the competencies to be gained by the students, leadership approaches used, administrative strategy, teaching faculty, student groups, partnership approaches, learning facilities to aid learning, application system, communication strategy, acculturation, financial support, mode of delivery, teaching, and assessment methods used. Appendix 1 summarizes the details of the practical steps taken under each of the framework domains. This was uniform across all four participating institutions.

### Study population

The participants of this study included faculty that had conducted training for the students at various institutions and the students who participated in the pilot implemented with the IPECP-IEs framework developed.

### Sampling method and recruitment of participants

Purposive sampling was used to identify study participants. This was used because of the nature of the study being qualitative and requiring us to get the best-fit participants to gain a deeper insight into the study objectives. We had 16 participants to sample from. All eligible participants who did not have time for an hour interview session and failed to provide consent were excluded. We only excluded four participants who were faculty based on the exclusion criteria. Each of the eligible participants was contacted via email for their interest to participate in the study. Upon acceptance, an online consent form and online Zoom link were sent to them. As we recruited the participants to enable us to establish saturation, we analyzed the data immediately after the KII. Saturation was reached at eight KIIs i.e., five KIIs with the students and three KIIs with the faculty. To confirm saturation, four additional KIIs i.e., one from faculty and three from students were conducted. As a result, twelve participants i.e., four faculty and eight students were recruited. While the point of saturation determined the number of participants in this study, efforts to ensure equal representation from all the institutions were made. Table 1 shows the equal distribution of participants with representation from the four institutions. It is important to note that the faculty and students who participated in the piloting of the IPECP-IEs framework were not involved in the development of the framework. This was done to control for bias.

### Study tools

A Key Informant Interview (KII) guide adapted from the SWOT analysis guide to developing and implementing IPECP programs in Health Care Education at Academic Medical Centers developed by Topor et al., 2018 was used [18]. The KII guide was modified to match the international electives learning environment and pre-tested by the research team to suit this study in another AFREhealth institution which was not part of the four main institutions that participated in this study. The KII guide used is attached as Appendix 2. This had both open and closed-ended questions. The closed-ended questions were followed by an open-ended question to allow an in-depth description by the participant. The KII guide used included a section on socio-demographic characteristics if the framework used is feasible and enabled learning, strengths, weakness, opportunities, and threats. The section on the strengths of the framework was a multiple-choice question that allowed each participant to respond to the component of the framework that they thought was a strength. This was followed by an open-ended question to enable the participants to describe their perspectives on the strengths in depth.

### Data collection

Participants were approached via email, thereafter, consent forms and interview guides were emailed to the participants at least 2 days before the interview to enable substantive preparation. The KIIs were conducted online via Zoom. This was in consideration of the COVID-19 situation but also much more cost-effective to collect the data from the various African countries. Each interview lasted one hour. Participant responses from the KIIs were audio-recorded and later transcribed verbatim. The interviewer took field notes. During data collection, items on the leadership approach and the multilateral collaboration approach were more easily answered by the faculty than by the students. However, a detailed explanation was done to ensure the students got to understand each structure in-depth.

### Data analysis

Deductive thematic analysis was employed in which Atlas Ti version 8 software was used. Major themes were already predetermined as strengths, weaknesses, opportunities, and threats to the framework. With the predetermined major themes, reading the transcripts several times to identify meaningful units and texts in line with the major themes was done. The texts were then condensed and coded. The codes generated from the transcripts were categorized as per the major themes and results reported. The socio-demographic characteristics of the participants were summarized and presented as frequencies. Furthermore, since the responses on strengths of the framework had both open and closed-end questions, the closed-ended responses were summarized and presented as frequencies, followed by the deductive thematic analysis of the open-ended portion.

### Quality control

The trustworthiness and rigor of this study given its qualitative nature were observed. For credibility, prolonged engagement of the participants during the interview and having a six-week duration of the IEs, having the research team review the findings, and double data analysis by having the results be analyzed by the lead author and a qualitative analysis expert was done. Furthermore, the collection of data from different participants and institutions was done to ensure triangulation. Given the different categories of participants i.e., students and faculty, efforts were made to ensure a detailed understanding of the questions by the students, especially on the questions on leadership and partnership approach. This was done by defining what the two meant and how they were implemented at their institution. A detailed description of the qualitative data collection and analysis process was done to ensure transferability in similar contexts elsewhere. To observe the dependability of the findings statistical software was used to derive findings. To observe confirmability the study findings were reviewed by a qualitative data analysis expert and shared with the study team for accuracy and alignment with the study objectives.

## Results

### Participant characteristics

As shown in Table 1, there was an equal distribution and representation from each institution among the participants. The disciplines represented in this table are not the only disciplines represented at the participating institutions. But rather, these are the disciplines that are accepted to participate and thus meet the criteria of IPECP which emphasizes two or more disciplines.

**Table 1:** Socio-demographic characteristics of the key informant interview participants. N = 12

Characteristic	Frequency ( N)
**Institution / Country Location**	
Kenyatta University, Kenya	3 − 2 Students (1 male, 1 female) 1 Academic (Male)
Makerere University, Uganda	3 − 2 Students (1 male, 1 female) 1 Academic (Male)
University of Ibadan, Nigeria	3 − 2 Students (1 male, 1 female) 1 Academic (female)
University of Zimbabwe Nigeria	3 − 2 Students (1 male, 1 female) 1 Academic (female)
**Gender**	
Male	6
Female	6
**Role at Institution**	
Academic Faculty	4
Students	8 ( description of rotations provided in the next section)
**Professional Discipline**	
Medicine	4
Nursing	3
Pharmacy	3
Physiotherapy	2

### Students rotations

Since there were four institutions included rotations were done as follows. Students from Makerere University were attached to Kenyatta University, Kenya while the students from the University of Ibadan, Nigeria were attached to Kenyatta University, Kenya. Students from the University of Kenyata, Kenya were attached to the University of Ibadan Nigeria while the students from the University of Zimbabwe, Zimbabwe were attached to Makerere University, Uganda. The various topics each team focused on are exhibited in Appendix 1 under Sect. 11. The various approaches to teaching, learning, and assessment were similar and are described in the framework developed and attached as Appendix 2.

### Strength, weakness, opportunities, and threats of the IPECP-IEs framework used

Four themes were considered regarding the framework used to implement the IPECP-IEs pilot among four African health professional training institutions. All the constructs, domains, and sections in the framework are not merely theoretical but were used by each institution. These included: (1) strength of the framework, (2) Weakness of the framework, (3) Opportunities from the framework, and (4) Threats to the Framework used.

### Theme1. Strengths of the IPECP-IEs framework

From the findings of this study, it generally appeared that all participants perceived the framework used to implement the IPECP- IEs as useful and appropriate to enable them to gain the IPECP skills and objectives set. Table 2 shows the distribution of the faculty and student participants’ responses to various structures of the framework in line with the strength.

**Table 2:** Faculty and Student Participant Responses on the Strength of the various domains of the IPECP-IEs Framework. N = 12

Framework Domain	Students who thought it was a strength N = 8	Faculty who thought it was a strength N = 4	Total
Curriculum aims and objectives clearly communicated and achieved at the end of the IPECP-IEs	7	4	11
Home and Host institutional leadership support approaches to enable the implementation of the IPECP-IEs	8	4	12
Administration between home and host institutions that included MOUs, handling applications by students, regular communication on steps, guidelines on various stages before, during, and after the elective	8	4	12
Faculty adequately skilled to deliver and guide learning	8	4	12
Student teams, number, and discipline selected to participate in the IPECP-IEs	8	2	10
Multilateral partnership approach that allowed reciprocity with equal benefits for both home and host institutions	8	4	12
Learning facilities to aid learning included Zoom, voice-over power points, teaching plans, curriculum, and reference materials to aid learning	8	4	12
Web Application system to enable applications to enable centralization of the application and acceptance process	8	4	12
Communication strategy i.e., emails and WhatsApp groups between faculty and students pre, during, and post-elective participation	8	4	12
The teaching method used; country-specific case studies to guide learning and acquisition of IPECP	7	4	11
Assessment methods used student lead i.e., pre-, and post-knowledge and skills scale using the ICCASS 2018 revised, group assignments, and joint report submission about the IPECP-IEs	8	4	12
Acculturation process that involved online voice-over power points for the students and an online faculty workshop that allowed faculty to gain skills in IPECP competencies, teaching, assessment, and virtual teaching skills	8	4	12
Online virtual learning model utilizing both synchronous sessions and asynchronous sessions	6	4	10
Elective Duration involved one week of orientation and 6 weeks of attachment and learning at the host institution	4	4	8
Funding to facilitate the student’s internet connection, faculty internet and time compensation, and Institutional Administrative costs	8	4	12

Items on the leadership approach and the multilateral collaboration approach were more easily answered by the faculty than the students. However, a detailed explanation was done to ensure the students got to understand each structure in-depth

Upon analysis of the narrative responses to the strengths of the framework, all the participants (12) thought that the acculturation component that allowed the training of the faculty and students on the IPECP concepts and roles before participation was an added advantage. The communication strategy that included the creation of WhatsApp groups with faculty and students enabled team building through instant social communication. The country-specific case studies allowed students to learn and appreciate cultural differences and similarities while gaining IPECP skills. This is shown in the quotes below. Each quote is presented and then followed by the KII number and the type of participant in bold. Participants’ institutions are not included to observe confidentiality.

*We were oriented well as faculty, and we knew how to teach and assess the students since IPECP is seldom done in my institution-****KII 03 faculty comment***.

*For me, the communication strategy especially the WhatsApp groups was the winner. It allowed us to break hieratical barriers and interact with the faculty and the rest of the team. For sure our teamwork was strengthened by this-****KII 05 student comment***.

*It was amazing to see how the country-specific case study we used enabled me to understand more about the country and how the cultural practices influenced patient outcomes-****KII 02 Student comment***.

### Theme 2 weakness of the IPECP-IEs framework

Although all the students and faculty (12) reported having met all their learning objectives and outcomes set by the Framework., some weaknesses were mentioned. This included the duration of the elective attachment to the host institution (6 weeks) being short and needing to be increased, internet variability during the Zoom sessions, difficulty finding a common time to meet as different professional disciplines due to different curricula, and lack of a physical mobility component that allows students to visit the country. This is reflected in the following quotes. Each quote is presented and then followed by the KII number and the type of participant in bold. Participants’ institutions are not included to observe confidentiality.

*The virtual aspect could be improved by having a blended approach. I think it should have a component of real-time. When talking about issues facing the country, if you are in the country and interacting with the patient, you will have a better understanding of the context-****KII03 student comment***.

*We have different schedules because we are from different disciplines so we had difficulty harmonizing time, but we would eventually find time given the online nature* ***- KII04 student comment.***

*The internet connection and strength would sometimes fluctuate- KII05 student.*

*We need to increase the time framework, to two or more weeks from 6 weeks to 8–10 weeks so that we can interact more physically-****KII07 student comment***.

### Theme 3. Opportunities from the IPECP-IEs framework

The various opportunities that are presented included acting as a starting point to structure and implement IPECP across various training institutions in Africa, advance research, and networking opportunities to share the best practices. This is evidenced in the quotes below. Each quote is presented and then followed by the KII number and the type of participant in bold. Participants’ institutions are not included to observe confidentiality.

*Because we have already established this small group among four training institutions in Africa, maybe we could expand the initiative or strengthen it so that the students in this initiative can have more opportunities out of this framework like research-****KII 01 faculty comment.***

*I think there are many strengths and the fact that this program’s framework is working, we could widen it and make it wider to more African training institutions -****KII04 faculty comment***.

*The various contacts and relations we have built at the host institution can be used for future collaboration in various academic, practice, and research avenues-****KII 06 student comment***.

### Theme 4. Threats to the IPECP-IEs framework

One of the issues that were often mentioned is the perceived obstacles that would hinder the implementation of the framework in various training institutions. This included siloed training where the current training curriculum of the students does not have opportunities that allow the students to study with, from, and about each. This is reflected in the statement below. Each quote is presented and then followed by the KII number and the type of participant in bold. Participants’ institutions are not included to observe confidentiality.

*The issue is our curricula as different disciplines do not make us have a common time to meet and study together which is a threat. We had to use our vacation time to improve teamwork-****KII07 Student comment.***

## Discussion

This study aimed to explore the experiences of faculty and students regarding the use of the developed IPECP-IEs framework. This further focused on the strength, weaknesses, opportunities, and threats of the framework developed to guide the implementation of IPECP_IEs which was piloted among four African health professional training institutions. It is important to note that IEs can be delivered using the virtual, blended, or physical mobility mode of delivery. In all the later modes of delivery, to enable experiencing what is done in another country, learning should be guided and based on the host institution’s country. Given that this was a virtual mode of delivery that was used, learning was guided using country-specific case studies and the faculty of the host institution. This enabled learning and appreciation of the other countries’ context. The findings generally show that the framework used enabled the students to gain the IPECP objectives set while appreciating the transcultural differences and similarities in another country. It is also important to note that this pilot of the framework was conducted with a regional approach of partnerships among institutions in Africa. This approach has been used to implement physical mobility IEs amongst various African health professional training institutions [12] which enabled less time zone differences and the gaining of knowledge and skills applicable back home [19] given the similarity in disease trends and systems [20]. This study is among the few that is exhibiting the feasibility of IEs with a virtual mode of delivery with the regional mobility approach among African health professional training institutions.

Given that country-specific case studies were used to guide learning from the host institution, they are means of advancing internationalization in health professions training through IEs but also with added uniqueness to cultivate IPECP skills among learners. These findings are similar to the findings of Leathers et al. 2018 that also found that IEs were useful platforms to advance the acquisition of IPECP skills [21]. However, Leather et al. study was conducted through actual physical mobility while our approach was virtual. The ability of the framework to allow a provision of pre-orientation training to both faculty and students on the concepts of IPECP was an added strength given that many faculty and students in Africa have reported a gap in IPECP knowledge and skills [22]. It is key to note that the pre-orientation training was similar across the institutions and conducted at the same time for the students and the faculty, The student’s orientation was done online asynchronously using voice-over power points while that of the faculty was done synchronously via Zoom training workshop.

In this framework, the elective duration was 6 weeks of online active learning from the host institution and one week of doing the preorientation online course and the pre-knowledge assessment survey. Although all the faculty mentioned that the duration was adequate, some of the students (4) thought that it would be better if the duration was prolonged to about 8–10 weeks to enable them to have a physical experience by visiting the country itself, and thus appreciate the transcultural similarities and differences in the IPECP command in various countries much more. However, it is important to note that despite the preference being a blended approach by the students, the virtual approach enabled students to gain the IPECP skills as per the objectives set in the framework. This is also an example of how faculty and students’ perspectives can be different however, a blended approach has the added benefits of real-time experience which is not offered with a virtual approach. Furthermore, on our end, given that funding is often a hindrance to many African health professional training students participating in IEs [19], we see a cost-effectiveness strength in the virtual mode of delivery to IEs given that travel and accommodation costs are not inquired. However, just like in this framework, the virtual electives must be done using country-specific case studies and the faculty of the host institution to enable appreciation of what happens in another country. Nevertheless, virtual learning is becoming more common in the post-COVID-19 environment [23] even in Africa [24]. The virtual IEs approach is heavily reliant on the internet and thus, there were some difficulties in internet strength given that weekly synchronous sessions were happening on Zoom. However, this was dealt with by ensuring all students and faculty were given funds to enable them to subscribe to stronger internet than that at the institution which may be slowed down due to many users. These findings are similar to those of Storz 2022 where the COVID-19 environment changed the IEs learning programs implementation approaches, and virtual electives [25] are one of the approaches that have gained momentum.

Given that there has been a growing global interest in innovative approaches towards internationalization in health professions education [3], many institutions have resulted in IEs through various forms of partnerships among home and host institutions. This framework provides a starting point for benchmarking to structure IPECP during IEs given that most happen in silos with pairing of students from the same discipline. This is because the framework provides the IPECP skills to be gained, teaching methods, and assessment approaches among others. Furthermore, this framework’s implementation presents an opportunity to evidence that IEs can be a useful platform to advance IPECP yet are often not utilized by various training institutions [21]. It also presents a starting point to advance the harmonization of IPECP training in Africa among health professions training institutions given the leadership and faculty networks created which can be expanded.

Although the IPECP-IEs approach using a virtual mode of delivery seems to be feasible as exhibited in this study among various African health professional training institutions, one of the biggest threats that it faces is the fact that IPECP is not yet embedded in the curriculum of many health professional training institutions in Africa and beyond. As reported in this study, all professional disciplines study in silos and often do not have opportunities to learn with, from, and about each other in Interprofessional teams in clinical training. Although this framework ensured that both faculty and students are oriented to the IPECP domains before participation, there is a need for health professional training institutions to prioritize adding IPECP to the curriculum in the various health professional training programs. Furthermore, having continued professional development sessions for faculty in IPECP could be done through the health professions education departments at the various institutions [26].

This study was qualitative and thus prone to participant acquiescence bias. However, the research team ensured open-ended questions were part of the guide and provided enough time to provide in-depth responses with an emphasis on the correct understanding of the questions by the participants. The study was only conducted in four training universities in East, West, and Southern Africa, without including Northern African countries as they are in the Eastern Mediterranean Region (EMRO). However, the strength of these results is that they represent the context of Sub-Saharan Africa thus giving a wider scope of description relatable to the wider sub-Saharan Africa and other low and middle-income countries globally. Furthermore, the study has all-around feedback on the framework used for IPECP during IEs from the faculty and students who participated in its pilot and thus allowing experiential feedback as opposed to perceptions.

Overall, this study confirms that the framework used to implement IPECP- IEs is adaptable and enabled students to gain the IPECP skills and objectives that were set. The virtual approach used in this framework to implement the IPECP-IEs is feasible among the various African health professional training institutions. The findings in line with the weaknesses can be worked out by increasing the duration and ensuring the extra weeks if resources are available to enable physical mobility to allow a blended approach. This framework was piloted amongst African health professional training institutions. However, its approach and findings are relevant even beyond Africa given that structuring of the country-specific studies can be done in other institutions in other countries and regions.

## Conclusion

The framework developed to guide the implementation of IPECP- IEs is feasible and enabled students to achieve the IPECP objectives set while appreciating the transcultural similarities and differences in another country. However, an increment of the framework’s duration to 8–10 weeks to enable a blended approach with a physical mobility approach was recommended by the participants.

### Recommendations

We recommend more researchers in the field of internationalization of health professions education to pilot the framework used to enhance its validity. The results of this study reflect the virtual mode of delivery used to pilot the framework. It remains to be seen if the framework is implementable using the blended and physical mobility mode of delivery of IEs.

### Electronic supplementary material

Below is the link to the electronic supplementary material.

Supplementary Material 1

Supplementary Material 2

## Data Availability

Availability. The tools and data set for this study are available upon reasonable request from the corresponding author.
