# Supplementary material for: Experiences of faculty and students regarding a locally developed framework for implementing interprofessional education during international electives in Sub-Saharan Africa
Source: BMC Med Educ. 2023 Sep 26;23:702. doi: 10.1186/s12909-023-04664-9 (PMC10523611; doi:10.1186/s12909-023-04664-9)
Supplement: Supplementary file 1 — Supplementary Material 1 [file 12909_2023_4664_MOESM1_ESM.docx]

In bold are the different framework main domains and under each domain are the various practical steps taken (not in bold). In total there are 12 framework domains, and some domains have subdomains in italics. Under each domain are the item or practical steps taken in each domain

**Interprofessional Education and Collaborative Practice (IPECP) Competencies to be gained.**

| **Competencies to be gained by students participating in IPECP international electives.**  **By the end of the international elective students should be able to ;** |
| --- |
| Demonstrate Knowledge, Skills, and attitudes towards teamwork |
| Demonstrate knowledge and understanding of the different roles, boundaries, responsibilities, and expertise of various health professionals in the team |
| communicate effectively and respectfully with other health professionals’ students, faculty, patients, community, etc |
| Demonstrate an awareness of cultural differences in health profession command and conduct in another country |
| Express one's opinions with others involved in the team with respect and humility |
| Reflect critically and evaluate their performance and that of the team |
| Develop a plan on how to apply interprofessional education and skills gained during the international elective back home in the clinical, community, or public health setting |
| Recognize the central role of the patient/ community in collaborative care |
| Acknowledge the views and ideas of other professionals during the international elective |

**Implementation Guide for the Delivery of IPECP during International Electives (IEs) in Health Professions Education Institutions**

| 1. **Home and host institutional leadership support for IPECP in IEs programs** |
| --- |
| All institutional leadership was given an overview of the IPECP-IEs program |
| Endorsed the IPECP-IEs program, Signed MOUs/multilateral agreements, and led the program to the official institutional structure to support the program i.e., administration, faculty selection, etc. |
| Responded to emails throughout the program development and inception |
| 1. **Home and host institution administrative support to handle students' and faculty logistical needs before, during, and after the IE placement** |
| Each institution had a program manager or international office to administrate the program.  Each assigned handling person at home and host institution had an IPECP-IEs orientation overview meeting to enable a generic understanding of the program.  IPECP-IEs Program manager roles were to   - Review applications, consult with host faculty for application review, and then proceed with acceptance notice. - Provide guidelines on the student preparation checklist. (If blended and physical mobility, assist with providing guidance on visa applications, Institutional requirements, ground transport, airport pick up, etc) - If the program is virtual, provide an overview of the program's aims, teaching plan, learning materials, a platform to be used for learning, etc. - Provide an acculturation and orientation session to the students. - Develop a clear compensation plan for the faculty's time and logistical support to students. - Manage all financial implications with proper accountability and documentation. - All other roles that may arise |
| 1. **Faculty at the host institution to support and supervise students** |
| Two faculty from different health professions were selected to participate and facilitate training for the students. The selected faculty had the following qualifications:   - Interest and involvement in IPECP - Experience in supervising and supporting international students. - community placements and field supervision experience, - Prior IPECP teaching experience was an added advantage |
| 1. **Partnership agreements that explore and allow reciprocity with home and host institutions** |
| A unifying multilateral agreement that binds all schools participating in the IPECP-IEs program with well-defined roles for both home and host institutions with equal opportunities for both home and host institutions was used. |
| 1. **Learning Facilities to aid student learning** |
| **Virtual Interaction:** Internet connection, online learning platforms, elective curriculum, teaching plans, teaching, and learning materials, Zoom used for synchronous sessions |
| 1. **Clear application system in place to guide students on IPECP elective application requirements** |
| A web-based application system that gave a detailed overview of the electives available at each institution with the ability to allow students to upload supporting documents irrespective of their country location was designed and provided |
| A clear submission guide with the ability to identify when electives will be open was made available to the students |
| Extra requirements specific to IPECP, i.e., students needed to apply as a cohort from one home institution to another institution were given |
| 1. **Communication strategy between home and host institution during preparations, implementation, and post participation** |
| Regular email communications were used. Furthermore, WhatsApp groups were created between the host faculty and the students to enhance instant communication |
| 1. **Adequate financial support to cater to student's and Faculty's logistical costs** |
| Each institution received funds in the amount of 2800 USD to enable students to subscribe to better internet, compensate faculty time, and cater for institutional administrative costs. |
| 1. **The IPECP student groups during the elective placement at host institutions** |
| A cohort of five students per institution was selected from different disciplines and joined attached to a host institution. The various disciplines included medicine, nursing, pharmacy, physiotherapy, dentistry, and biomedical sciences.  In total twenty students participated in this program |
| 1. **Acculturation Considerations** |
| ***10.1Students' Pre-elective IPECP orientation didactic sessions or seminars offered by the host institution to students, to enable understanding of roles, expectations, the domains of IPE, and the flow of activities*** |
| Asynchronous sessions using voice-over power points were given to each student group. Similar material was given to all students to enable uniformity. These gave.   - An overview of the program, - Aims of the program, - Define IPECP, - Define students' roles and expectations as they participate, - The flow of activities, - Duration of elective |
| ***10.2 Pre-Elective IPECP training (workshops or seminars) offered to both home and host institution faculty, and clinical and community instructors, to enable understanding of roles, expectations, the domains of IPECP, and the flow of activities at home and host institutions*** |
| We conducted an online workshop with all faculty and institutional leaders of the participating institutions delivered by IPECP experts to enable them to understand:  The IPECP-IEs program (background, overview, aims, span, etc.)   - - The structure, acculturation, and principles for student participation   - Faculty roles   - IPECP definitions and core competencies   - The importance of IPECP in healthcare   - The various teaching methods for IPECP virtually   - The various learning assessment methods for IPECP virtually |
| ***10.3 Onsite Orientation by the host institution on various social aspects to enable the acclimatization of students in consideration of language, cultural humility, and equity.*** |
| - Since this was done online the first session with the faculty and program manager focussed on this at the host institution was done. Both the students and faculty were able to give an overview of their countries' culture, school culture, language preferences, way of life, etc.  - Continued interaction for team building was done through creating a WhatsApp group among the students and the host institution faculty, continued online interaction, and social communication groups throughout the program**.** |
| 1. **IPECP teaching approaches utilized during International Electives at Host institutions** |
| ***11.1 Country-Specific case study-based interprofessional teaching.***   - These extensively described the issue at hand and had some probe questions to stimulate student joint engagement, through understanding the issue and innovatively addressing the issue at hand as a team.   The case studies offered by each of the four institutions include.   1. COVID-19 pandemic challenges and hopes. A case study in Kenya at Kenyatta University, Kenya 2. Targeted maternal health initiatives for reducing maternal mortality and morbidity at Makerere University, Uganda 3. Cancer Diagnosis in Women and their Quality of Life in Nigeria at the University of Ibadan, Nigeria 4. Optimizing antiretroviral therapy adherence (O-ART) in Zimbabwe, At the University of Zimbabwe, Zimbabwe |
| 1. **IPECP learner's Assessment Approaches during international electives at Host Institutions** |
| ***12.1 Formative ( ongoing assessment )*** |
| **12.1.1 Pre-elective course Knowledge/Skills/ Attitudes Surveys**   - This was administered online using Microsoft Forms, This was based on the Interprofessional Collaborative Competency Attainment Scale (ICCAS 2018)[1]. |
| ***12.1.2Portfolio-based assessments***   - collection and review of individual and group work projects or assignments done weekly |
| **12.1.3 *Peer to Peer assessment***   - This was done during a joint session that was synchronous |
| ***12.2 Summative Assessment ( End of Program Assessment)*** |
| **12.2.1 Post Elective course knowledge/skills/attitude surveys.**   - This was administered online using Microsoft Forms, This was based on the Interprofessional Collaborative Competency Attainment Scale (ICCAS 2018)[1]. |
| **12.2.2 Self-reflection through Elective Report at the end**   - A uniform template was given to all student groups to enable each of the groups to jointly make a well-structured report in all domains of the entire rotation including IPECP skills gained |
| **12.2.3 Group feedback sessions**   - This was done online by having students jointly report on the IPECP gained their experiences, and their main takeaways with the aid of PowerPoint presentations at the end of their rotation. |
| 1. **Mode of Elective Delivery** |
| This was done online using synchronous and asynchronous sessions for 6 weeks. Synchronous sessions were conducted using Zoom once a week for 1-1.5 hours. These aimed at having report-back sessions on the group assignments, faculty lecture sessions, and clarification on any concepts. |
