# Supplementary material for: Experiences of faculty and students regarding a locally developed framework for implementing interprofessional education during international electives in Sub-Saharan Africa
Source: BMC Med Educ. 2023 Sep 26;23:702. doi: 10.1186/s12909-023-04664-9 (PMC10523611; doi:10.1186/s12909-023-04664-9)
Supplement: Supplementary file 2 — Supplementary Material 2 [file 12909_2023_4664_MOESM2_ESM.docx]

**Appendix 2:** **Faculty and student Experiences and Analysis of the developed IPECP_IEs Framework Survey**

This survey aims to establish the SWOT of the Interprofessional Education and Collaborative Practice (IPECP) framework used to implement the International Electives (IEs ) pilot program you participate in as a student or faculty.

1. KII Number
2. Name of your Institution

Makerere University

Kenyatta University

University of Ibadan

University of Zimbabwe

1. Designation in the program

Faculty

Student

1. Which professional Discipline do you belong to?
2. Years of service as faculty
3. Year of study if student
4. Age
5. what disciplines of students were in the cohort you participated in?

9. What was the name of the elective you participated in during the IPECP_IEs?

10. Which country did it focus on?

11. How many student members were in the team you worked with?

**IPECP_IES Framework SWOT Analysis**

12. What was done well ( strengths) during the IEs guided by an IPECP framework to ensure the effective acquisition of IPECP skills in an international elective learning environment? ( Select all that apply)

Program Orientation/ Acculturation

Administrative support

The curriculum aims and objectives clarity.

Teaching faculty available with enough skills to guide the learning.

Leaning methods used

The technology used ( virtual or real-time)

Assessment methods

Logistical support and funding

Elective duration

Host and Home institution relationships

Gaining a deeper understanding of IPE/ IPC competencies

Understanding the importance of IPEC in healthcare

Understanding the importance of IPEC in improving patient outcomes

13. Please explain your choices

13. What needs to be enhanced ( weakness) to increase the success and use of the developed IPECP_IEs framework?

14. What other internal or external factors place this IPECP-IEs framework used at a disadvantage?

15. What external factors could benefit this IPECP-IEs framework? *

16. How can the strengths of this framework be used to take advantage of opportunities for this pilot program?

17. How can we minimize/overcome weaknesses by using opportunities? *

18. What factors beyond your control place the developed IPECP_IES framework you used to guide your learning “at risk?”

19. How can you use the framework's strengths to minimize threats? *

20. How can you use threats (hindrances) to minimize weaknesses and use weaknesses to avoid threats?

21. What model of the elective did you utilize?

Online ( virtual mobility)

22. Was the virtual model utilized enabling to learn IPECP skills despite the COVID-19 pandemic?

Yes

No

Please explain your response

23. Should this IPECP framework for International electives be used and adopted by various institutions?

Yes

No

Please explain your response

24. Below are the various IPECP skills the Framework aimed to have the learners achieve. Which of the following were achieved? *

Demonstrate Knowledge and attitudes, and skills for, teamwork.

Demonstrate knowledge and understanding of the different roles, boundaries, responsibilities, and expertise of various health professionals in the team.

communicate effectively and respectfully with other health professionals’ students, faculty, patients, community, etc.

Demonstrate an awareness of cultural differences in health profession command and conduct in another country.

Express one's opinions with others involved in patient care with respect and humility.

Reflect critically and evaluate their performance and that of the team.

Develop a plan on how to apply interprofessional education and skills gained during the international elective back home in the clinical, community, or public health setting.

Recognize the central role of the patient/ community in collaborative care.

Acknowledge the views and ideas of other professionals during an international elective.
